# Supplementary material for: Hot spot prediction in protein-protein interactions by an ensemble system
Source: BMC Syst Biol. 2018 Dec 31;12(Suppl 9):132. doi: 10.1186/s12918-018-0665-8 (PMC6311905; doi:10.1186/s12918-018-0665-8)
Supplement: Supplementary file 2 — All relevant MATLAB code. A simple Matlab implement of our predictor. (ZIP 19,742 kb) [file 12918_2018_665_MOESM2_ESM.zip › Additional file 2/Read ASA/ASA website/dbMPIKT/4KRPA.html]

 NetSurfP - Protein Surface Accessibility and Secondary Structure Predictions

  

|  |  |  |
| --- | --- | --- |
|  |  | NetSurfP - Protein Surface Accessibility and Secondary Structure Predictions  Technical University of Denmark |

  


---

```
# For publication of results, please cite:
# A generic method for assignment of reliability scores applied to solvent accessibility predictions.
# Bent Petersen, Thomas Nordahl Petersen, Pernille Andersen, Morten Nielsen and Claus Lundegaard
# BMC Structural Biology 2009, 9:51 doi:10.1186/1472-6807-9-51
#
# Column 1: Class assignment - B for buried or E for Exposed - Threshold: 25% exposure, but not based on RSA
# Column 2: Amino acid
# Column 3: Sequence name
# Column 4: Amino acid number
# Column 5: Relative Surface Accessibility - RSA
# Column 6: Absolute Surface Accessibility
# Column 7: Z-fit score for RSA prediction
# Column 8: Probability for Alpha-Helix
# Column 9: Probability for Beta-strand
# Column 10: Probability for Coil
E L  4KRPA                  1    0.685 125.497  -0.710   0.003   0.003   0.994
E E  4KRPA                  2    0.604 105.554  -0.262   0.018   0.047   0.935
E E  4KRPA                  3    0.662 115.669   0.726   0.018   0.047   0.935
E K  4KRPA                  4    0.487 100.258   0.040   0.018   0.088   0.893
E K  4KRPA                  5    0.500 102.871   1.164   0.020   0.205   0.775
E V  4KRPA                  6    0.257  39.578   0.515   0.021   0.451   0.528
B C  4KRPA                  7    0.054   7.525  -0.557   0.021   0.451   0.528
E Q  4KRPA                  8    0.509  90.854   0.759   0.021   0.451   0.528
B G  4KRPA                  9    0.180  14.150  -4.123   0.021   0.279   0.699
E T  4KRPA                 10    0.372  51.583  -0.359   0.019   0.141   0.840
E S  4KRPA                 11    0.571  66.909  -1.110   0.018   0.047   0.935
E N  4KRPA                 12    0.394  57.608  -1.799   0.018   0.047   0.935
E K  4KRPA                 13    0.493 101.369  -1.280   0.018   0.088   0.893
B L  4KRPA                 14    0.261  47.807  -1.491   0.020   0.205   0.775
B T  4KRPA                 15    0.157  21.845  -2.161   0.019   0.141   0.840
E Q  4KRPA                 16    0.464  82.853  -1.044   0.019   0.141   0.840
B L  4KRPA                 17    0.266  48.796  -2.663   0.018   0.047   0.935
E G  4KRPA                 18    0.368  28.938  -1.844   0.018   0.019   0.964
E T  4KRPA                 19    0.409  56.756  -0.951   0.058   0.017   0.925
B F  4KRPA                 20    0.281  56.397  -0.883   0.339   0.016   0.645
E E  4KRPA                 21    0.509  88.887  -0.575   0.354   0.048   0.598
E D  4KRPA                 22    0.369  53.101  -1.258   0.268   0.043   0.689
E H  4KRPA                 23    0.289  52.587   0.416   0.522   0.016   0.462
B F  4KRPA                 24    0.189  38.013   0.231   0.879   0.010   0.111
E L  4KRPA                 25    0.550 100.760   0.816   0.858   0.002   0.139
E S  4KRPA                 26    0.395  46.353   0.247   0.923   0.002   0.076
B L  4KRPA                 27    0.073  13.421  -0.350   0.923   0.002   0.076
E Q  4KRPA                 28    0.302  53.991   0.695   0.923   0.002   0.076
E R  4KRPA                 29    0.525 120.271   0.802   0.858   0.002   0.139
E M  4KRPA                 30    0.262  52.426   0.844   0.782   0.003   0.216
B F  4KRPA                 31    0.068  13.547  -0.028   0.430   0.016   0.555
E N  4KRPA                 32    0.548  80.286   1.201   0.181   0.016   0.803
E N  4KRPA                 33    0.577  84.473   0.172   0.018   0.019   0.964
B C  4KRPA                 34    0.045   6.360   0.875   0.004   0.197   0.799
E E  4KRPA                 35    0.308  53.773   0.508   0.003   0.718   0.279
B V  4KRPA                 36    0.241  37.103   1.345   0.001   0.959   0.040
B V  4KRPA                 37    0.081  12.465   0.764   0.001   0.959   0.040
B L  4KRPA                 38    0.328  60.020  -0.109   0.002   0.816   0.182
B G  4KRPA                 39    0.100   7.886  -1.175   0.003   0.718   0.279
B N  4KRPA                 40    0.256  37.552  -0.153   0.002   0.816   0.182
B L  4KRPA                 41    0.060  11.004   0.681   0.001   0.900   0.099
B E  4KRPA                 42    0.207  36.076   0.377   0.001   0.959   0.040
B I  4KRPA                 43    0.033   6.049   0.547   0.001   0.959   0.040
B T  4KRPA                 44    0.109  15.132   0.933   0.001   0.900   0.099
B Y  4KRPA                 45    0.115  24.490   0.152   0.002   0.816   0.182
B V  4KRPA                 46    0.052   7.946   0.224   0.004   0.514   0.481
E Q  4KRPA                 47    0.454  81.013  -0.320   0.019   0.141   0.840
E R  4KRPA                 48    0.545 124.782  -1.110   0.018   0.047   0.935
B N  4KRPA                 49    0.274  40.172  -0.956   0.018   0.047   0.935
E Y  4KRPA                 50    0.451  96.421  -1.093   0.018   0.019   0.964
E D  4KRPA                 51    0.551  79.341  -0.965   0.018   0.019   0.964
B L  4KRPA                 52    0.154  28.216  -0.879   0.018   0.047   0.935
E S  4KRPA                 53    0.496  58.143  -1.351   0.053   0.043   0.903
B F  4KRPA                 54    0.243  48.850  -0.011   0.053   0.043   0.903
B L  4KRPA                 55    0.115  21.148  -0.487   0.058   0.017   0.925
E K  4KRPA                 56    0.561 115.377   0.416   0.053   0.043   0.903
E T  4KRPA                 57    0.441  61.139   0.499   0.113   0.087   0.800
B I  4KRPA                 58    0.106  19.573   0.472   0.066   0.296   0.638
E Q  4KRPA                 59    0.341  60.849   0.472   0.087   0.683   0.230
E E  4KRPA                 60    0.287  50.104   0.181   0.021   0.756   0.223
B V  4KRPA                 61    0.082  12.573   0.510   0.018   0.846   0.136
B A  4KRPA                 62    0.249  27.462   0.176   0.023   0.655   0.322
B G  4KRPA                 63    0.119   9.397  -1.047   0.023   0.655   0.322
B Y  4KRPA                 64    0.081  17.288   0.565   0.018   0.846   0.136
B V  4KRPA                 65    0.032   4.857   0.153   0.011   0.918   0.071
B L  4KRPA                 66    0.054   9.961   0.832   0.011   0.918   0.071
B I  4KRPA                 67    0.027   4.940   0.555   0.018   0.846   0.136
B A  4KRPA                 68    0.083   9.202  -0.549   0.021   0.756   0.223
B L  4KRPA                 69    0.139  25.414  -0.295   0.022   0.552   0.426
B N  4KRPA                 70    0.205  30.056  -0.532   0.020   0.205   0.775
E T  4KRPA                 71    0.429  59.572  -0.465   0.018   0.088   0.893
B V  4KRPA                 72    0.117  17.998  -0.671   0.018   0.088   0.893
E E  4KRPA                 73    0.618 108.000  -0.011   0.019   0.141   0.840
E R  4KRPA                 74    0.500 114.523   0.094   0.020   0.205   0.775
B I  4KRPA                 75    0.175  32.449   0.972   0.020   0.205   0.775
E P  4KRPA                 76    0.466  66.097  -0.401   0.018   0.088   0.893
B L  4KRPA                 77    0.105  19.134  -0.302   0.018   0.047   0.935
E E  4KRPA                 78    0.527  91.980  -0.140   0.018   0.019   0.964
E N  4KRPA                 79    0.452  66.231  -0.056   0.018   0.047   0.935
B L  4KRPA                 80    0.072  13.128   0.590   0.022   0.359   0.619
E Q  4KRPA                 81    0.333  59.438   0.671   0.018   0.846   0.136
E I  4KRPA                 82    0.263  48.599   0.267   0.011   0.918   0.071
B I  4KRPA                 83    0.080  14.856   0.852   0.018   0.846   0.136
B R  4KRPA                 84    0.311  71.196   0.479   0.023   0.655   0.322
B G  4KRPA                 85    0.181  14.284  -2.364   0.020   0.205   0.775
E N  4KRPA                 86    0.394  57.696   0.707   0.069   0.386   0.545
E M  4KRPA                 87    0.468  93.607   0.462   0.023   0.655   0.322
B Y  4KRPA                 88    0.219  46.736   0.389   0.079   0.592   0.329
B Y  4KRPA                 89    0.201  43.018   0.174   0.069   0.386   0.545
E E  4KRPA                 90    0.538  93.936  -0.955   0.056   0.142   0.802
E N  4KRPA                 91    0.519  75.982  -1.127   0.052   0.084   0.864
E S  4KRPA                 92    0.412  48.310   0.212   0.064   0.216   0.721
B Y  4KRPA                 93    0.145  30.986   0.110   0.021   0.451   0.528
B A  4KRPA                 94    0.030   3.361  -0.069   0.021   0.756   0.223
B L  4KRPA                 95    0.032   5.841   0.050   0.011   0.918   0.071
B A  4KRPA                 96    0.029   3.207   0.190   0.006   0.962   0.032
B V  4KRPA                 97    0.018   2.767   0.969   0.006   0.962   0.032
B L  4KRPA                 98    0.030   5.530   0.114   0.006   0.962   0.032
B S  4KRPA                 99    0.064   7.536  -0.986   0.006   0.962   0.032
B N  4KRPA                100    0.083  12.137  -0.386   0.018   0.846   0.136
B Y  4KRPA                101    0.202  43.189  -0.169   0.004   0.514   0.481
E D  4KRPA                102    0.424  61.113  -0.636   0.005   0.262   0.733
E A  4KRPA                103    0.591  65.095  -1.631   0.005   0.045   0.951
E N  4KRPA                104    0.615  90.051  -2.156   0.005   0.015   0.979
E K  4KRPA                105    0.457  93.923  -0.957   0.018   0.047   0.935
E T  4KRPA                106    0.340  47.200  -1.471   0.018   0.088   0.893
E G  4KRPA                107    0.310  24.366  -0.276   0.022   0.359   0.619
B L  4KRPA                108    0.144  26.311  -0.456   0.023   0.655   0.322
E K  4KRPA                109    0.308  63.273  -0.030   0.021   0.756   0.223
E E  4KRPA                110    0.364  63.556  -0.084   0.018   0.846   0.136
B L  4KRPA                111    0.053   9.759   1.320   0.021   0.756   0.223
E P  4KRPA                112    0.392  55.667   0.043   0.004   0.616   0.381
B M  4KRPA                113    0.047   9.405  -0.133   0.005   0.336   0.660
E R  4KRPA                114    0.422  96.546  -0.103   0.004   0.138   0.858
E N  4KRPA                115    0.566  82.789  -0.621   0.018   0.047   0.935
B L  4KRPA                116    0.242  44.310  -0.888   0.056   0.142   0.802
E Q  4KRPA                117    0.297  53.116  -0.159   0.021   0.451   0.528
E E  4KRPA                118    0.339  59.241   0.214   0.021   0.756   0.223
B I  4KRPA                119    0.080  14.819   0.247   0.018   0.846   0.136
B L  4KRPA                120    0.217  39.714  -0.346   0.023   0.655   0.322
E H  4KRPA                121    0.467  84.947  -0.739   0.004   0.420   0.576
B G  4KRPA                122    0.243  19.148  -0.445   0.004   0.197   0.799
B A  4KRPA                123    0.136  15.031  -0.810   0.004   0.420   0.576
B V  4KRPA                124    0.042   6.486  -0.998   0.001   0.959   0.040
B R  4KRPA                125    0.172  39.388   1.611   0.000   0.983   0.017
B F  4KRPA                126    0.030   5.981  -0.051   0.000   0.983   0.017
B S  4KRPA                127    0.116  13.619  -0.103   0.001   0.959   0.040
E N  4KRPA                128    0.363  53.158  -0.389   0.003   0.718   0.279
B N  4KRPA                129    0.084  12.342  -0.553   0.004   0.197   0.799
E P  4KRPA                130    0.550  78.088  -0.730   0.018   0.088   0.893
E A  4KRPA                131    0.500  55.089  -1.030   0.018   0.088   0.893
B L  4KRPA                132    0.224  40.978  -1.670   0.019   0.141   0.840
B C  4KRPA                133    0.264  37.009  -1.702   0.019   0.141   0.840
E N  4KRPA                134    0.508  74.430  -1.581   0.018   0.088   0.893
E V  4KRPA                135    0.340  52.319  -1.159   0.018   0.047   0.935
E E  4KRPA                136    0.270  47.134  -1.111   0.018   0.047   0.935
E S  4KRPA                137    0.433  50.724  -0.556   0.184   0.043   0.773
E I  4KRPA                138    0.469  86.784  -1.398   0.257   0.016   0.727
E Q  4KRPA                139    0.375  67.011  -0.633   0.257   0.016   0.727
B W  4KRPA                140    0.092  22.150  -0.468   0.600   0.003   0.397
E R  4KRPA                141    0.526 120.546   0.921   0.622   0.015   0.363
E D  4KRPA                142    0.357  51.473   0.544   0.717   0.014   0.269
B I  4KRPA                143    0.055  10.119  -0.379   0.622   0.015   0.363
B V  4KRPA                144    0.179  27.512  -0.604   0.561   0.047   0.393
E S  4KRPA                145    0.642  75.184   0.629   0.354   0.048   0.598
E S  4KRPA                146    0.449  52.611  -0.752   0.018   0.019   0.964
E D  4KRPA                147    0.690  99.415  -1.159   0.005   0.015   0.979
E F  4KRPA                148    0.469  94.229  -1.518   0.005   0.045   0.951
E L  4KRPA                149    0.440  80.564  -0.630   0.004   0.085   0.910
E S  4KRPA                150    0.494  57.873  -1.206   0.004   0.138   0.858
E N  4KRPA                151    0.489  71.634  -0.914   0.005   0.336   0.660
B M  4KRPA                152    0.210  41.961  -0.558   0.004   0.420   0.576
E S  4KRPA                153    0.445  52.131  -1.665   0.022   0.359   0.619
E M  4KRPA                154    0.398  79.640  -1.746   0.022   0.359   0.619
E D  4KRPA                155    0.475  68.390  -1.207   0.021   0.451   0.528
E F  4KRPA                156    0.342  68.639  -1.449   0.021   0.451   0.528
E Q  4KRPA                157    0.483  86.335  -1.403   0.021   0.279   0.699
E N  4KRPA                158    0.451  65.968  -1.907   0.018   0.088   0.893
E H  4KRPA                159    0.667 121.327  -1.379   0.018   0.047   0.935
E L  4KRPA                160    0.654 119.711  -1.763   0.005   0.045   0.951
E G  4KRPA                161    0.447  35.171  -0.049   0.004   0.138   0.858
E S  4KRPA                162    0.565  66.171   0.561   0.005   0.336   0.660
B C  4KRPA                163    0.128  17.971  -1.310   0.005   0.262   0.733
E Q  4KRPA                164    0.546  97.551  -0.119   0.004   0.197   0.799
E K  4KRPA                165    0.564 116.056   0.441   0.019   0.141   0.840
B C  4KRPA                166    0.071   9.996  -1.896   0.018   0.047   0.935
E D  4KRPA                167    0.543  78.203  -0.524   0.018   0.047   0.935
E P  4KRPA                168    0.712 101.004  -0.842   0.018   0.047   0.935
E S  4KRPA                169    0.481  56.361  -0.098   0.018   0.047   0.935
B C  4KRPA                170    0.177  24.851  -0.704   0.018   0.088   0.893
E P  4KRPA                171    0.592  83.962  -1.153   0.004   0.085   0.910
E N  4KRPA                172    0.646  94.574  -0.183   0.004   0.138   0.858
E G  4KRPA                173    0.260  20.478  -1.081   0.005   0.262   0.733
E S  4KRPA                174    0.438  51.322  -0.453   0.004   0.420   0.576
B C  4KRPA                175    0.165  23.180  -2.577   0.022   0.552   0.426
E W  4KRPA                176    0.546 131.409  -1.278   0.004   0.420   0.576
E G  4KRPA                177    0.451  35.517  -2.068   0.004   0.085   0.910
E A  4KRPA                178    0.659  72.589  -1.481   0.018   0.047   0.935
E G  4KRPA                179    0.508  39.995  -1.108   0.004   0.085   0.910
E E  4KRPA                180    0.386  67.452  -0.561   0.018   0.088   0.893
E E  4KRPA                181    0.592 103.405  -0.852   0.019   0.141   0.840
E N  4KRPA                182    0.442  64.680   0.493   0.021   0.279   0.699
B C  4KRPA                183    0.100  14.054  -3.039   0.021   0.279   0.699
E Q  4KRPA                184    0.428  76.352  -0.596   0.022   0.552   0.426
E K  4KRPA                185    0.427  87.916  -0.968   0.021   0.451   0.528
B L  4KRPA                186    0.200  36.583  -0.535   0.021   0.279   0.699
E T  4KRPA                187    0.454  62.998  -0.916   0.020   0.205   0.775
E K  4KRPA                188    0.580 119.306  -0.991   0.019   0.141   0.840
E I  4KRPA                189    0.426  78.884  -1.371   0.019   0.141   0.840
E I  4KRPA                190    0.418  77.256  -0.858   0.021   0.451   0.528
B C  4KRPA                191    0.208  29.175  -0.988   0.021   0.451   0.528
E A  4KRPA                192    0.372  41.005  -1.044   0.022   0.359   0.619
E Q  4KRPA                193    0.587 104.802  -1.675   0.020   0.205   0.775
E Q  4KRPA                194    0.512  91.407  -0.545   0.019   0.141   0.840
B C  4KRPA                195    0.122  17.185  -1.577   0.018   0.047   0.935
E S  4KRPA                196    0.493  57.744  -1.156   0.018   0.047   0.935
E G  4KRPA                197    0.630  49.589  -2.074   0.018   0.088   0.893
E R  4KRPA                198    0.491 112.416  -1.742   0.021   0.279   0.699
B C  4KRPA                199    0.231  32.432  -1.211   0.022   0.359   0.619
E R  4KRPA                200    0.420  96.066  -1.308   0.021   0.451   0.528
E G  4KRPA                201    0.331  26.089  -1.384   0.021   0.279   0.699
E K  4KRPA                202    0.594 122.104  -1.919   0.019   0.141   0.840
E S  4KRPA                203    0.545  63.851  -1.572   0.018   0.047   0.935
E P  4KRPA                204    0.618  87.751  -0.811   0.018   0.047   0.935
E S  4KRPA                205    0.473  55.447  -0.813   0.018   0.047   0.935
E D  4KRPA                206    0.485  69.845  -0.141   0.018   0.088   0.893
E C  4KRPA                207    0.402  56.483   0.331   0.020   0.205   0.775
B C  4KRPA                208    0.157  22.015  -0.446   0.020   0.205   0.775
B H  4KRPA                209    0.212  38.526   0.048   0.056   0.142   0.802
E N  4KRPA                210    0.610  89.319  -0.369   0.053   0.043   0.903
E Q  4KRPA                211    0.470  83.996   0.743   0.053   0.043   0.903
B C  4KRPA                212    0.032   4.563  -1.304   0.053   0.043   0.903
E A  4KRPA                213    0.411  45.325  -0.349   0.052   0.084   0.864
E A  4KRPA                214    0.595  65.624  -0.854   0.053   0.043   0.903
E G  4KRPA                215    0.414  32.613  -0.052   0.018   0.088   0.893
B C  4KRPA                216    0.122  17.101  -2.025   0.020   0.205   0.775
E T  4KRPA                217    0.548  75.980  -1.480   0.004   0.197   0.799
E G  4KRPA                218    0.446  35.077  -0.856   0.005   0.045   0.951
E P  4KRPA                219    0.527  74.852  -1.258   0.005   0.015   0.979
E R  4KRPA                220    0.556 127.278  -0.446   0.003   0.003   0.994
E E  4KRPA                221    0.501  87.490  -0.374   0.016   0.005   0.979
E S  4KRPA                222    0.664  77.797  -0.338   0.018   0.019   0.964
E D  4KRPA                223    0.404  58.260   1.361   0.018   0.047   0.935
B C  4KRPA                224    0.042   5.911  -0.671   0.064   0.216   0.721
E L  4KRPA                225    0.312  57.036   0.511   0.021   0.451   0.528
E V  4KRPA                226    0.430  66.091   0.492   0.022   0.552   0.426
B C  4KRPA                227    0.066   9.224  -0.567   0.022   0.359   0.619
E R  4KRPA                228    0.449 102.867  -0.124   0.066   0.296   0.638
E K  4KRPA                229    0.439  90.220  -0.572   0.020   0.205   0.775
B F  4KRPA                230    0.276  55.433  -0.701   0.021   0.279   0.699
E R  4KRPA                231    0.418  95.630  -0.807   0.020   0.205   0.775
E D  4KRPA                232    0.467  67.266  -1.184   0.019   0.141   0.840
E E  4KRPA                233    0.665 116.158  -1.370   0.005   0.015   0.979
E A  4KRPA                234    0.570  62.792  -1.711   0.005   0.015   0.979
E T  4KRPA                235    0.498  69.017   0.681   0.004   0.138   0.858
B C  4KRPA                236    0.089  12.468  -1.306   0.004   0.514   0.481
E K  4KRPA                237    0.391  80.449   0.101   0.023   0.655   0.322
E D  4KRPA                238    0.351  50.507  -1.177   0.023   0.655   0.322
E T  4KRPA                239    0.509  70.571   0.005   0.022   0.359   0.619
B C  4KRPA                240    0.068   9.477  -0.201   0.004   0.085   0.910
E P  4KRPA                241    0.441  62.592  -0.731   0.005   0.015   0.979
E P  4KRPA                242    0.670  95.030  -0.805   0.018   0.019   0.964
E L  4KRPA                243    0.457  83.603  -2.068   0.019   0.141   0.840
B M  4KRPA                244    0.299  59.830  -0.831   0.021   0.451   0.528
B L  4KRPA                245    0.242  44.255  -1.357   0.023   0.655   0.322
E Y  4KRPA                246    0.417  89.134  -1.111   0.022   0.552   0.426
E N  4KRPA                247    0.346  50.654  -1.463   0.005   0.262   0.733
E P  4KRPA                248    0.669  94.917  -1.672   0.005   0.045   0.951
E T  4KRPA                249    0.673  93.317  -1.216   0.004   0.085   0.910
E T  4KRPA                250    0.336  46.562  -0.235   0.004   0.420   0.576
E Y  4KRPA                251    0.249  53.211  -0.243   0.004   0.616   0.381
E Q  4KRPA                252    0.409  73.101   0.630   0.018   0.846   0.136
B M  4KRPA                253    0.107  21.411  -1.609   0.021   0.756   0.223
E D  4KRPA                254    0.449  64.658  -0.370   0.023   0.655   0.322
E V  4KRPA                255    0.545  83.828  -0.954   0.021   0.279   0.699
B N  4KRPA                256    0.220  32.135  -0.478   0.018   0.019   0.964
E P  4KRPA                257    0.629  89.184  -0.884   0.018   0.019   0.964
E E  4KRPA                258    0.636 111.074  -0.787   0.018   0.047   0.935
E G  4KRPA                259    0.475  37.343  -0.361   0.019   0.141   0.840
E K  4KRPA                260    0.257  52.844  -1.031   0.022   0.359   0.619
E Y  4KRPA                261    0.356  76.099  -0.256   0.022   0.359   0.619
E S  4KRPA                262    0.432  50.572  -1.460   0.020   0.205   0.775
E F  4KRPA                263    0.418  83.812  -1.099   0.004   0.085   0.910
E G  4KRPA                264    0.641  50.447  -1.661   0.005   0.015   0.979
E A  4KRPA                265    0.531  58.472  -0.994   0.003   0.003   0.994
E T  4KRPA                266    0.633  87.853   0.214   0.018   0.088   0.893
E C  4KRPA                267    0.244  34.244  -0.444   0.021   0.279   0.699
B V  4KRPA                268    0.202  31.001   0.020   0.022   0.359   0.619
E K  4KRPA                269    0.499 102.685  -0.285   0.022   0.359   0.619
E K  4KRPA                270    0.467  96.041   0.262   0.020   0.205   0.775
B C  4KRPA                271    0.069   9.758  -0.470   0.005   0.045   0.951
E P  4KRPA                272    0.547  77.563  -0.616   0.005   0.015   0.979
E R  4KRPA                273    0.713 163.392  -0.889   0.005   0.045   0.951
E N  4KRPA                274    0.466  68.208  -1.455   0.018   0.088   0.893
B Y  4KRPA                275    0.234  49.942  -0.433   0.021   0.279   0.699
B V  4KRPA                276    0.281  43.128  -0.980   0.021   0.451   0.528
B V  4KRPA                277    0.197  30.217  -1.312   0.022   0.552   0.426
E T  4KRPA                278    0.351  48.642  -0.985   0.021   0.451   0.528
E D  4KRPA                279    0.574  82.656  -2.129   0.019   0.141   0.840
E H  4KRPA                280    0.663 120.563  -1.522   0.005   0.045   0.951
E G  4KRPA                281    0.474  37.288  -0.552   0.005   0.045   0.951
E S  4KRPA                282    0.426  49.986   0.143   0.021   0.279   0.699
B C  4KRPA                283    0.103  14.517  -1.590   0.022   0.552   0.426
E V  4KRPA                284    0.307  47.155  -0.553   0.023   0.655   0.322
E R  4KRPA                285    0.366  83.768  -0.919   0.022   0.552   0.426
E A  4KRPA                286    0.563  62.076  -0.341   0.022   0.359   0.619
B C  4KRPA                287    0.086  12.018  -1.089   0.004   0.085   0.910
E G  4KRPA                288    0.526  41.388  -1.080   0.005   0.015   0.979
E A  4KRPA                289    0.662  72.908  -1.441   0.005   0.015   0.979
E D  4KRPA                290    0.538  77.540  -2.116   0.018   0.047   0.935
E S  4KRPA                291    0.283  33.168  -0.896   0.021   0.279   0.699
E Y  4KRPA                292    0.292  62.315  -0.835   0.023   0.655   0.322
E E  4KRPA                293    0.417  72.797  -0.674   0.023   0.655   0.322
E M  4KRPA                294    0.249  49.825  -1.491   0.022   0.552   0.426
E E  4KRPA                295    0.570  99.649  -1.793   0.005   0.262   0.733
E E  4KRPA                296    0.727 127.024  -1.488   0.005   0.045   0.951
E D  4KRPA                297    0.695 100.178  -0.595   0.005   0.015   0.979
E G  4KRPA                298    0.495  38.949  -0.309   0.018   0.019   0.964
E V  4KRPA                299    0.381  58.606   0.333   0.019   0.141   0.840
E R  4KRPA                300    0.440 100.714   0.288   0.021   0.451   0.528
E K  4KRPA                301    0.469  96.494   1.534   0.022   0.552   0.426
B C  4KRPA                302    0.051   7.160  -1.828   0.022   0.552   0.426
E K  4KRPA                303    0.498 102.356   0.822   0.023   0.655   0.322
E K  4KRPA                304    0.545 112.148   0.598   0.022   0.359   0.619
B C  4KRPA                305    0.083  11.667  -2.039   0.019   0.141   0.840
E E  4KRPA                306    0.550  96.137  -1.018   0.020   0.205   0.775
E G  4KRPA                307    0.516  40.578  -1.076   0.020   0.205   0.775
E P  4KRPA                308    0.494  70.070  -1.193   0.019   0.141   0.840
B C  4KRPA                309    0.167  23.489  -2.102   0.021   0.279   0.699
E R  4KRPA                310    0.508 116.424  -1.133   0.021   0.451   0.528
E K  4KRPA                311    0.437  89.850  -1.305   0.021   0.451   0.528
E V  4KRPA                312    0.386  59.297  -1.603   0.021   0.451   0.528
E C  4KRPA                313    0.469  65.820  -1.090   0.022   0.359   0.619
E N  4KRPA                314    0.562  82.335  -1.405   0.020   0.205   0.775
E G  4KRPA                315    0.603  47.464  -1.792   0.018   0.088   0.893
E I  4KRPA                316    0.466  86.265  -1.414   0.019   0.141   0.840
E G  4KRPA                317    0.551  43.372  -1.198   0.021   0.279   0.699
E I  4KRPA                318    0.285  52.669  -1.500   0.022   0.359   0.619
E G  4KRPA                319    0.464  36.509  -1.273   0.021   0.451   0.528
E E  4KRPA                320    0.360  62.979  -1.364   0.022   0.552   0.426
B F  4KRPA                321    0.213  42.669  -1.767   0.022   0.359   0.619
E K  4KRPA                322    0.454  93.450  -2.006   0.021   0.279   0.699
E D  4KRPA                323    0.471  67.857  -1.874   0.021   0.279   0.699
E S  4KRPA                324    0.396  46.446  -1.488   0.022   0.359   0.619
E L  4KRPA                325    0.323  59.160  -1.699   0.022   0.359   0.619
E S  4KRPA                326    0.313  36.707  -1.925   0.022   0.359   0.619
B I  4KRPA                327    0.217  40.182  -1.251   0.021   0.279   0.699
E N  4KRPA                328    0.441  64.518  -1.250   0.021   0.279   0.699
E A  4KRPA                329    0.380  41.865  -1.564   0.019   0.141   0.840
E T  4KRPA                330    0.464  64.357  -2.379   0.020   0.205   0.775
E N  4KRPA                331    0.452  66.246  -1.827   0.021   0.279   0.699
B I  4KRPA                332    0.234  43.272  -2.204   0.021   0.279   0.699
E K  4KRPA                333    0.485  99.847  -1.417   0.021   0.279   0.699
E H  4KRPA                334    0.325  59.136  -1.495   0.021   0.279   0.699
E F  4KRPA                335    0.426  85.498  -1.570   0.019   0.141   0.840
E K  4KRPA                336    0.596 122.535  -1.439   0.004   0.085   0.910
E N  4KRPA                337    0.576  84.312  -0.465   0.004   0.085   0.910
B C  4KRPA                338    0.089  12.468  -1.853   0.004   0.197   0.799
E T  4KRPA                339    0.480  66.562  -0.419   0.003   0.718   0.279
E S  4KRPA                340    0.412  48.240   0.140   0.002   0.816   0.182
B I  4KRPA                341    0.168  31.117  -0.765   0.002   0.816   0.182
E S  4KRPA                342    0.533  62.421  -1.599   0.004   0.514   0.481
E G  4KRPA                343    0.442  34.825  -1.945   0.004   0.197   0.799
E D  4KRPA                344    0.491  70.695  -1.739   0.005   0.336   0.660
B L  4KRPA                345    0.246  45.043  -0.890   0.004   0.616   0.381
E H  4KRPA                346    0.437  79.508  -1.116   0.021   0.756   0.223
B I  4KRPA                347    0.185  34.206  -2.248   0.023   0.655   0.322
E L  4KRPA                348    0.383  70.127  -1.253   0.022   0.359   0.619
B P  4KRPA                349    0.303  42.996  -1.672   0.021   0.279   0.699
E V  4KRPA                350    0.598  91.959  -2.106   0.019   0.141   0.840
E A  4KRPA                351    0.494  54.472  -1.721   0.020   0.205   0.775
B F  4KRPA                352    0.217  43.552  -1.612   0.021   0.451   0.528
E R  4KRPA                353    0.544 124.668  -1.441   0.022   0.359   0.619
E G  4KRPA                354    0.523  41.144  -2.052   0.019   0.141   0.840
E D  4KRPA                355    0.606  87.339  -2.152   0.005   0.045   0.951
B S  4KRPA                356    0.266  31.175  -2.186   0.005   0.045   0.951
E F  4KRPA                357    0.473  94.891  -1.579   0.004   0.085   0.910
E T  4KRPA                358    0.548  76.021  -1.233   0.004   0.197   0.799
E H  4KRPA                359    0.387  70.486  -1.757   0.004   0.197   0.799
E T  4KRPA                360    0.429  59.544  -1.715   0.004   0.085   0.910
E P  4KRPA                361    0.482  68.353  -1.683   0.018   0.047   0.935
E P  4KRPA                362    0.446  63.358  -1.411   0.018   0.019   0.964
E L  4KRPA                363    0.361  66.136  -1.355   0.058   0.017   0.925
E D  4KRPA                364    0.463  66.747  -0.798   0.181   0.016   0.803
E P  4KRPA                365    0.501  71.035  -1.090   0.257   0.016   0.727
E Q  4KRPA                366    0.451  80.477  -0.354   0.268   0.043   0.689
B E  4KRPA                367    0.234  40.862  -1.098   0.307   0.165   0.527
E L  4KRPA                368    0.416  76.133  -0.511   0.278   0.093   0.628
E D  4KRPA                369    0.642  92.512  -0.183   0.278   0.093   0.628
E I  4KRPA                370    0.295  54.649  -1.131   0.184   0.043   0.773
E L  4KRPA                371    0.349  63.957  -1.226   0.058   0.017   0.925
E K  4KRPA                372    0.604 124.263  -1.189   0.018   0.019   0.964
E T  4KRPA                373    0.471  65.369   0.285   0.018   0.088   0.893
B V  4KRPA                374    0.144  22.148  -0.389   0.005   0.336   0.660
E K  4KRPA                375    0.448  92.215  -0.069   0.004   0.616   0.381
E E  4KRPA                376    0.522  91.106   0.218   0.003   0.718   0.279
B I  4KRPA                377    0.111  20.627  -0.997   0.004   0.616   0.381
E T  4KRPA                378    0.480  66.562  -1.467   0.005   0.336   0.660
E G  4KRPA                379    0.475  37.359  -1.574   0.004   0.138   0.858
E F  4KRPA                380    0.416  83.531  -1.614   0.004   0.197   0.799
B L  4KRPA                381    0.235  42.974  -1.382   0.021   0.451   0.528
E L  4KRPA                382    0.454  83.054  -0.739   0.022   0.552   0.426
B I  4KRPA                383    0.183  33.892  -1.985   0.022   0.552   0.426
E Q  4KRPA                384    0.498  88.871  -1.290   0.021   0.451   0.528
E A  4KRPA                385    0.483  53.249  -1.585   0.022   0.359   0.619
E W  4KRPA                386    0.267  64.117  -1.048   0.020   0.205   0.775
E P  4KRPA                387    0.474  67.232  -1.200   0.018   0.088   0.893
E E  4KRPA                388    0.538  93.936  -1.510   0.053   0.043   0.903
E N  4KRPA                389    0.583  85.366  -1.095   0.053   0.043   0.903
B R  4KRPA                390    0.261  59.655  -1.609   0.053   0.043   0.903
E T  4KRPA                391    0.499  69.197  -1.550   0.018   0.047   0.935
E D  4KRPA                392    0.469  67.554  -1.915   0.018   0.088   0.893
E L  4KRPA                393    0.381  69.706  -0.832   0.019   0.141   0.840
E H  4KRPA                394    0.398  72.469  -1.537   0.020   0.205   0.775
B A  4KRPA                395    0.257  28.343  -1.831   0.021   0.279   0.699
B F  4KRPA                396    0.147  29.463  -2.134   0.019   0.141   0.840
E E  4KRPA                397    0.570  99.649  -0.708   0.019   0.141   0.840
E N  4KRPA                398    0.578  84.648  -1.413   0.018   0.088   0.893
E L  4KRPA                399    0.298  54.619  -0.946   0.018   0.088   0.893
E E  4KRPA                400    0.522  91.106  -0.245   0.005   0.336   0.660
E I  4KRPA                401    0.278  51.393  -0.028   0.004   0.616   0.381
E I  4KRPA                402    0.271  50.153  -0.902   0.003   0.718   0.279
E R  4KRPA                403    0.512 117.362  -1.238   0.023   0.655   0.322
E G  4KRPA                404    0.475  37.343  -1.448   0.021   0.451   0.528
E R  4KRPA                405    0.430  98.562  -0.551   0.022   0.552   0.426
E T  4KRPA                406    0.461  63.899  -0.742   0.022   0.552   0.426
E K  4KRPA                407    0.288  59.159  -0.376   0.022   0.359   0.619
E Q  4KRPA                408    0.472  84.299  -1.079   0.020   0.205   0.775
E H  4KRPA                409    0.647 117.635  -2.137   0.018   0.088   0.893
E G  4KRPA                410    0.597  46.976  -2.280   0.018   0.047   0.935
E Q  4KRPA                411    0.442  78.959  -1.401   0.020   0.205   0.775
B F  4KRPA                412    0.247  49.513  -1.288   0.022   0.359   0.619
B S  4KRPA                413    0.236  27.671  -2.572   0.022   0.552   0.426
B L  4KRPA                414    0.177  32.372  -1.713   0.021   0.756   0.223
B A  4KRPA                415    0.234  25.754  -1.528   0.018   0.846   0.136
B V  4KRPA                416    0.183  28.096  -1.691   0.021   0.756   0.223
E V  4KRPA                417    0.506  77.695  -1.958   0.023   0.655   0.322
E S  4KRPA                418    0.431  50.501  -2.130   0.020   0.205   0.775
E L  4KRPA                419    0.465  85.123  -1.416   0.018   0.047   0.935
E N  4KRPA                420    0.493  72.117  -0.896   0.018   0.088   0.893
B I  4KRPA                421    0.161  29.785  -1.507   0.020   0.205   0.775
E T  4KRPA                422    0.409  56.701  -0.720   0.022   0.359   0.619
E S  4KRPA                423    0.451  52.869  -0.637   0.069   0.386   0.545
E L  4KRPA                424    0.306  56.084  -0.985   0.066   0.296   0.638
E G  4KRPA                425    0.399  31.370  -1.507   0.056   0.142   0.802
E L  4KRPA                426    0.436  79.850  -1.469   0.018   0.047   0.935
E R  4KRPA                427    0.612 140.148  -1.268   0.018   0.019   0.964
E S  4KRPA                428    0.588  68.902  -1.324   0.018   0.047   0.935
E L  4KRPA                429    0.335  61.339  -0.384   0.019   0.141   0.840
E K  4KRPA                430    0.390  80.285  -0.985   0.020   0.205   0.775
E E  4KRPA                431    0.368  64.220  -0.768   0.021   0.451   0.528
B I  4KRPA                432    0.105  19.333  -1.001   0.021   0.451   0.528
E S  4KRPA                433    0.449  52.646  -1.155   0.022   0.359   0.619
E D  4KRPA                434    0.510  73.520  -1.413   0.004   0.197   0.799
E G  4KRPA                435    0.387  30.481  -2.195   0.004   0.085   0.910
E D  4KRPA                436    0.460  66.243  -1.490   0.004   0.197   0.799
B V  4KRPA                437    0.234  36.027  -1.071   0.004   0.616   0.381
B I  4KRPA                438    0.219  40.589  -0.908   0.018   0.846   0.136
B I  4KRPA                439    0.187  34.558  -1.149   0.021   0.756   0.223
B S  4KRPA                440    0.344  40.305  -1.477   0.022   0.552   0.426
E G  4KRPA                441    0.421  33.164  -1.614   0.021   0.279   0.699
B N  4KRPA                442    0.183  26.762  -2.055   0.018   0.047   0.935
E K  4KRPA                443    0.379  77.899  -1.811   0.018   0.088   0.893
E N  4KRPA                444    0.531  77.738  -1.949   0.019   0.141   0.840
B L  4KRPA                445    0.264  48.265  -0.778   0.020   0.205   0.775
B C  4KRPA                446    0.193  27.041  -1.860   0.021   0.279   0.699
E Y  4KRPA                447    0.393  84.070  -1.260   0.022   0.359   0.619
E A  4KRPA                448    0.407  44.895  -1.770   0.022   0.359   0.619
E N  4KRPA                449    0.472  69.130  -1.584   0.021   0.279   0.699
E T  4KRPA                450    0.433  60.085  -2.073   0.020   0.205   0.775
E I  4KRPA                451    0.348  64.325  -1.411   0.019   0.141   0.840
E N  4KRPA                452    0.512  74.957  -0.873   0.018   0.088   0.893
E W  4KRPA                453    0.302  72.631  -0.986   0.018   0.088   0.893
E K  4KRPA                454    0.565 116.200  -1.614   0.019   0.141   0.840
E K  4KRPA                455    0.520 107.046  -1.174   0.020   0.205   0.775
E L  4KRPA                456    0.301  55.205  -1.109   0.022   0.359   0.619
E F  4KRPA                457    0.434  87.164  -1.560   0.021   0.451   0.528
E G  4KRPA                458    0.409  32.204  -0.693   0.021   0.451   0.528
E T  4KRPA                459    0.457  63.344  -1.143   0.020   0.205   0.775
E S  4KRPA                460    0.498  58.354  -1.303   0.018   0.088   0.893
E G  4KRPA                461    0.620  48.763  -1.794   0.005   0.045   0.951
E Q  4KRPA                462    0.531  94.854  -0.767   0.004   0.085   0.910
E K  4KRPA                463    0.592 121.774  -0.338   0.004   0.197   0.799
B T  4KRPA                464    0.176  24.411  -2.044   0.022   0.359   0.619
E K  4KRPA                465    0.464  95.424  -0.608   0.021   0.451   0.528
E I  4KRPA                466    0.276  51.005  -1.560   0.021   0.451   0.528
E I  4KRPA                467    0.491  90.872  -1.378   0.021   0.451   0.528
E S  4KRPA                468    0.408  47.876  -1.463   0.021   0.451   0.528
E N  4KRPA                469    0.348  50.976  -0.885   0.021   0.279   0.699
E R  4KRPA                470    0.521 119.401  -0.597   0.018   0.088   0.893
E G  4KRPA                471    0.462  36.344  -0.675   0.018   0.047   0.935
E E  4KRPA                472    0.533  93.098  -0.505   0.018   0.047   0.935
E N  4KRPA                473    0.567  82.980  -1.235   0.019   0.141   0.840
E S  4KRPA                474    0.473  55.436  -0.271   0.020   0.205   0.775
E C  4KRPA                475    0.306  43.005  -1.624   0.021   0.279   0.699
E K  4KRPA                476    0.548 112.826  -0.366   0.020   0.205   0.775
E A  4KRPA                477    0.545  60.070  -0.606   0.020   0.205   0.775
E T  4KRPA                478    0.465  64.482   0.329   0.020   0.205   0.775
E G  4KRPA                479    0.386  30.370  -1.271   0.021   0.279   0.699
E Q  4KRPA                480    0.384  68.654  -0.774   0.022   0.359   0.619
E V  4KRPA                481    0.428  65.814  -1.490   0.022   0.359   0.619
E C  4KRPA                482    0.314  44.128  -1.284   0.021   0.279   0.699
E H  4KRPA                483    0.485  88.149  -1.194   0.020   0.205   0.775
E A  4KRPA                484    0.347  38.184  -1.829   0.020   0.205   0.775
E L  4KRPA                485    0.590 107.937  -1.255   0.019   0.141   0.840
B C  4KRPA                486    0.133  18.687  -2.591   0.018   0.088   0.893
E S  4KRPA                487    0.601  70.414  -0.611   0.018   0.088   0.893
E P  4KRPA                488    0.431  61.173  -0.960   0.020   0.205   0.775
E E  4KRPA                489    0.581 101.571  -0.976   0.020   0.205   0.775
E G  4KRPA                490    0.500  39.350  -0.311   0.004   0.138   0.858
B C  4KRPA                491    0.106  14.812  -2.966   0.004   0.138   0.858
E W  4KRPA                492    0.532 127.826  -0.878   0.004   0.085   0.910
E G  4KRPA                493    0.405  31.881  -1.224   0.005   0.015   0.979
E P  4KRPA                494    0.695  98.592  -1.369   0.016   0.005   0.979
E E  4KRPA                495    0.616 107.580  -0.817   0.016   0.005   0.979
E P  4KRPA                496    0.349  49.580  -0.434   0.018   0.019   0.964
E R  4KRPA                497    0.551 126.293  -0.466   0.018   0.047   0.935
E D  4KRPA                498    0.357  51.516   0.632   0.019   0.141   0.840
B C  4KRPA                499    0.078  10.993  -2.626   0.021   0.279   0.699
E V  4KRPA                500    0.306  47.094  -0.582   0.021   0.451   0.528
E S  4KRPA                501    0.419  49.119  -0.550   0.021   0.451   0.528
B C  4KRPA                502    0.092  12.931  -1.086   0.022   0.359   0.619
E R  4KRPA                503    0.495 113.401  -0.829   0.021   0.279   0.699
E N  4KRPA                504    0.504  73.815  -1.119   0.020   0.205   0.775
E V  4KRPA                505    0.274  42.175  -1.497   0.019   0.141   0.840
E S  4KRPA                506    0.433  50.689  -1.357   0.019   0.141   0.840
E R  4KRPA                507    0.531 121.622  -1.056   0.019   0.141   0.840
E G  4KRPA                508    0.574  45.150  -1.012   0.018   0.088   0.893
E R  4KRPA                509    0.443 101.378  -0.697   0.005   0.045   0.951
E E  4KRPA                510    0.479  83.629  -0.025   0.020   0.205   0.775
B C  4KRPA                511    0.151  21.257  -1.504   0.022   0.359   0.619
E V  4KRPA                512    0.388  59.620  -0.476   0.022   0.359   0.619
E D  4KRPA                513    0.556  80.062  -1.199   0.021   0.279   0.699
E K  4KRPA                514    0.518 106.532   0.396   0.020   0.205   0.775
B C  4KRPA                515    0.041   5.700  -2.702   0.018   0.088   0.893
E N  4KRPA                516    0.518  75.821  -1.192   0.004   0.085   0.910
E L  4KRPA                517    0.477  87.339  -1.750   0.004   0.085   0.910
E L  4KRPA                518    0.495  90.598  -1.723   0.004   0.085   0.910
E E  4KRPA                519    0.485  84.712  -1.967   0.004   0.085   0.910
E G  4KRPA                520    0.457  35.982  -1.868   0.004   0.085   0.910
E E  4KRPA                521    0.541  94.478  -1.496   0.018   0.047   0.935
E P  4KRPA                522    0.484  68.708  -1.189   0.020   0.205   0.775
E R  4KRPA                523    0.392  89.768  -0.813   0.021   0.451   0.528
E E  4KRPA                524    0.381  66.578  -1.020   0.023   0.655   0.322
E F  4KRPA                525    0.223  44.716  -1.460   0.022   0.552   0.426
E V  4KRPA                526    0.348  53.518  -1.578   0.021   0.279   0.699
E E  4KRPA                527    0.685 119.600  -1.026   0.004   0.085   0.910
E N  4KRPA                528    0.716 104.764  -0.143   0.003   0.003   0.994
E S  4KRPA                529    0.481  56.373   0.437   0.018   0.019   0.964
E E  4KRPA                530    0.452  78.929   1.556   0.022   0.359   0.619
B C  4KRPA                531    0.087  12.215  -0.669   0.003   0.718   0.279
E I  4KRPA                532    0.400  74.000   0.997   0.021   0.756   0.223
E Q  4KRPA                533    0.524  93.569   0.861   0.022   0.552   0.426
B C  4KRPA                534    0.084  11.780  -0.412   0.019   0.141   0.840
E H  4KRPA                535    0.433  78.672   0.493   0.053   0.043   0.903
E P  4KRPA                536    0.628  89.127   0.172   0.115   0.016   0.868
E E  4KRPA                537    0.505  88.258   0.605   0.113   0.087   0.800
B C  4KRPA                538    0.047   6.669  -1.598   0.056   0.142   0.802
E L  4KRPA                539    0.448  82.029   0.136   0.064   0.216   0.721
E P  4KRPA                540    0.553  78.428  -0.610   0.056   0.142   0.802
E Q  4KRPA                541    0.345  61.546  -1.118   0.018   0.088   0.893
E A  4KRPA                542    0.491  54.086  -1.791   0.018   0.088   0.893
E M  4KRPA                543    0.566 113.177  -1.045   0.018   0.088   0.893
E N  4KRPA                544    0.513  75.059  -0.691   0.018   0.088   0.893
E I  4KRPA                545    0.536  99.178  -0.779   0.018   0.088   0.893
E T  4KRPA                546    0.407  56.437  -0.485   0.018   0.088   0.893
B C  4KRPA                547    0.113  15.935  -3.318   0.019   0.141   0.840
E T  4KRPA                548    0.566  78.476  -0.606   0.019   0.141   0.840
E G  4KRPA                549    0.446  35.061  -1.784   0.005   0.015   0.979
E R  4KRPA                550    0.597 136.599  -1.655   0.005   0.015   0.979
E G  4KRPA                551    0.564  44.371  -0.603   0.005   0.015   0.979
E P  4KRPA                552    0.368  52.219   0.043   0.018   0.019   0.964
E D  4KRPA                553    0.661  95.236  -0.654   0.018   0.019   0.964
E N  4KRPA                554    0.415  60.683   0.977   0.053   0.043   0.903
B C  4KRPA                555    0.071   9.982  -2.083   0.056   0.142   0.802
E I  4KRPA                556    0.342  63.214  -0.295   0.066   0.296   0.638
E Q  4KRPA                557    0.557  99.498   0.136   0.069   0.386   0.545
B C  4KRPA                558    0.142  19.951  -1.003   0.021   0.279   0.699
E A  4KRPA                559    0.438  48.290  -0.480   0.021   0.279   0.699
E H  4KRPA                560    0.446  81.036  -0.934   0.021   0.279   0.699
B Y  4KRPA                561    0.189  40.432  -0.338   0.022   0.359   0.619
E I  4KRPA                562    0.367  67.932  -1.082   0.020   0.205   0.775
E D  4KRPA                563    0.436  62.828  -1.296   0.018   0.088   0.893
E G  4KRPA                564    0.633  49.817  -1.164   0.018   0.019   0.964
E P  4KRPA                565    0.590  83.792  -0.998   0.005   0.015   0.979
E H  4KRPA                566    0.478  86.948   0.698   0.022   0.359   0.619
B C  4KRPA                567    0.060   8.396  -1.670   0.023   0.655   0.322
E V  4KRPA                568    0.343  52.765   0.572   0.023   0.655   0.322
E K  4KRPA                569    0.457  93.964  -0.365   0.023   0.655   0.322
E T  4KRPA                570    0.512  70.987   1.240   0.022   0.359   0.619
B C  4KRPA                571    0.054   7.638   0.016   0.005   0.015   0.979
E P  4KRPA                572    0.510  72.341  -0.136   0.003   0.003   0.994
E A  4KRPA                573    0.775  85.372  -0.092   0.003   0.003   0.994
E G  4KRPA                574    0.484  38.122  -1.597   0.018   0.088   0.893
B V  4KRPA                575    0.264  40.638  -0.340   0.004   0.420   0.576
E M  4KRPA                576    0.281  56.168  -0.631   0.003   0.718   0.279
E G  4KRPA                577    0.477  37.564  -0.677   0.021   0.756   0.223
E E  4KRPA                578    0.326  56.917  -1.576   0.021   0.451   0.528
E N  4KRPA                579    0.652  95.482  -1.536   0.018   0.088   0.893
E N  4KRPA                580    0.604  88.396  -1.610   0.019   0.141   0.840
E T  4KRPA                581    0.475  65.841  -0.209   0.022   0.359   0.619
E L  4KRPA                582    0.329  60.240  -0.658   0.023   0.655   0.322
E V  4KRPA                583    0.382  58.790  -0.749   0.023   0.655   0.322
E W  4KRPA                584    0.339  81.505  -1.007   0.023   0.655   0.322
E K  4KRPA                585    0.524 107.725  -0.683   0.022   0.552   0.426
E Y  4KRPA                586    0.383  81.911  -0.838   0.022   0.359   0.619
B A  4KRPA                587    0.225  24.806  -1.706   0.018   0.088   0.893
E D  4KRPA                588    0.527  75.926  -1.494   0.018   0.047   0.935
E A  4KRPA                589    0.627  69.106  -0.329   0.018   0.019   0.964
E G  4KRPA                590    0.644  50.699  -0.547   0.003   0.003   0.994
E H  4KRPA                591    0.503  91.532   0.284   0.005   0.015   0.979
E V  4KRPA                592    0.448  68.827   1.803   0.020   0.205   0.775
B C  4KRPA                593    0.048   6.725  -1.321   0.004   0.514   0.481
E H  4KRPA                594    0.411  74.815   1.239   0.004   0.616   0.381
E L  4KRPA                595    0.580 106.271   1.361   0.021   0.451   0.528
B C  4KRPA                596    0.124  17.424   0.184   0.018   0.088   0.893
E H  4KRPA                597    0.529  96.152   0.667   0.018   0.019   0.964
E P  4KRPA                598    0.695  98.606   0.133   0.016   0.005   0.979
E N  4KRPA                599    0.523  76.611   0.778   0.018   0.019   0.964
B C  4KRPA                600    0.050   6.964  -0.156   0.052   0.084   0.864
E T  4KRPA                601    0.501  69.461   1.023   0.066   0.296   0.638
E Y  4KRPA                602    0.475 101.593   0.656   0.074   0.484   0.442
E G  4KRPA                603    0.383  30.181  -0.062   0.021   0.451   0.528
B C  4KRPA                604    0.066   9.294  -1.019   0.021   0.451   0.528
E T  4KRPA                605    0.597  82.818  -0.318   0.005   0.336   0.660
E G  4KRPA                606    0.364  28.655  -1.016   0.004   0.138   0.858
E P  4KRPA                607    0.532  75.477  -1.497   0.005   0.015   0.979
E G  4KRPA                608    0.588  46.315  -0.225   0.005   0.015   0.979
E L  4KRPA                609    0.498  91.202  -0.619   0.018   0.019   0.964
E E  4KRPA                610    0.613 107.178   0.046   0.018   0.019   0.964
E G  4KRPA                611    0.504  39.625   1.076   0.018   0.047   0.935
B C  4KRPA                612    0.060   8.382  -0.746   0.021   0.279   0.699
E P  4KRPA                613    0.316  44.897   0.669   0.023   0.655   0.322
E T  4KRPA                614    0.495  68.726   1.455   0.021   0.756   0.223
B T  4KRPA                615    0.051   7.060   0.401   0.022   0.552   0.426
E N  4KRPA                616    0.556  81.384   0.329   0.019   0.141   0.840
E G  4KRPA                617    0.663  52.194  -0.043   0.018   0.019   0.964
E P  4KRPA                618    0.441  62.606  -1.635   0.018   0.019   0.964
E K  4KRPA                619    0.352  72.509  -0.349   0.020   0.205   0.775
B H  4KRPA                620    0.249  45.275  -0.112   0.023   0.655   0.322
B H  4KRPA                621    0.187  34.015   0.360   0.021   0.756   0.223
B H  4KRPA                622    0.240  43.620   0.017   0.018   0.846   0.136
B H  4KRPA                623    0.238  43.292  -0.719   0.021   0.756   0.223
E H  4KRPA                624    0.379  68.867   0.686   0.022   0.552   0.426
E H  4KRPA                625    0.773 140.591  -2.045   0.003   0.003   0.994

Explain the output.  Go back.
```

---
